# Mapping the Spread of Malaria Drug Resistance

**DOI:** 10.1371/journal.pmed.1000054

**Published:** 2009-04-14

**Authors:** Tim Anderson

**Affiliations:** Department of Genetics, Southwest Foundation for Biomedical Research, San Antonio, Texas, United States of America

## Abstract

Tim Anderson discusses a new study of molecular variation in alleles at the dihydropteroate synthase locus, which underlies resistance to sulfadoxine, in over 5,000 parasites from 50 locations.

**Linked Research Article**

This Perspective discusses the following new study published in *PLoS Medicine*:

Pearce RJ, Pota H, Evehe M-SB, Bâ E-H, Mombo-Ngoma G, et al. (2009) Multiple origins and regional dispersal of resistant *dhps* in African *Plasmodium falciparum* malaria. PLoS Med 6(4): e1000055.  doi:10.1371/journal.pmed.1000055

Cally Roper and colleagues analyze the distribution of sulfadoxine resistance mutations and flanking microsatellite loci to trace the emergence and dispersal of drug-resistant *Plasmodium falciparum* malaria in Africa.

Drug resistance is a recurrent theme in the history of infectious disease control. In the case of malaria, resistance to all but one of the five major classes of drugs is widespread [1]. Such resistance occurs because of the strong selection pressure associated with giving patients antimalarial drugs.

The most effective way to stall resistance would therefore be to eliminate selection by halting drug treatment [2], but this is rarely a feasible option. Hence alternative approaches to managing resistance are needed. First of all, we need to better understand the manner in which resistance evolves and spreads within populations.

Molecular methods provide the tools needed for investigating the evolution and the spread of resistance genes. These methods can be used to answer a multitude of pertinent questions: Do resistance alleles have few or many origins? Do they spread locally or globally? Do parasites form a single pan-African population, or are there barriers to gene flow? In turn, the answers to these questions can be used to make rational decisions about drug treatment policy.

## Sulfadoxine Resistance

Sulfadoxine (SDX) forms one half of the drug combination sulfadoxine-pyrimethamine (Fansidar), which replaced chloroquine as the first-line drug in much of Africa, before itself being replaced by artemisinin combination therapies. Resistance to both SDX and pyrimethamine (PYR) has spread rapidly within Africa. Because the genetic basis of resistance to these drugs is well established—single point mutations within the enzyme active sites [3]—SDX and PYR treatment provide excellent opportunities for understanding resistance evolution.

In a new study in *PLoS Medicine*, Cally Roper and colleagues describe molecular variation in alleles at the dihydropteroate synthase (*dhps*) locus, which is known to underlie resistance to SDX, in over 5,000 parasites from 50 locations during 2000–2007 [4]. They also examine sequence variation flanking this locus to determine the number of independent origins of resistance alleles. This unusually large data set provides an extraordinarily fine-grained view of the spread of resistance alleles across Africa.

## Curious Distribution of Resistance Alleles in East and West Africa

The maps generated by Roper and colleagues show a striking distribution of *dhps* alleles in Africa. Parasites bearing amino acids SGE (underlining denotes amino acids conferring resistance) at codons 436, 437, and 540 in *dhps* are common in east Africa, while parasites bearing AGK and SGK alleles are common in west Africa. The demonstration (below) that each one of these alleles has multiple origins suggests the action of selection. However, there are no obvious differences in treatment or biology of malaria in east and west Africa that explain repeated origins of dissimilar alleles. An alternative explanation is that the same alleles have arisen on multiple occasions because they were present within parasite populations at low levels prior to initiation of SDX-PYR selection.

## How Many Times Have Resistance Alleles Arisen?

The data from flanking microsatellite markers reveal that there have been a minimum of five independent origins of resistance, which show strong geographical clustering. SGE alleles have arisen at least twice in the east, with one lineage in Ethiopia and Sudan and the other spanning from Kenya to South Africa. In west Africa, there are three lineages of alleles; in each lineage both AGK and SGK are found on a common background, suggesting that one has given rise to the other. Once again, the three AGK/SGK lineages have a strikingly focal distribution; lineage 1 is found from Namibia to Gabon, lineage 2 in Cameroon, and lineage 3 from Nigeria to Senegal.

How can we explain this focal distribution of allelic lineages? One possibility is that resistance alleles have spread like ripples on a pond from a limited number of foci. In this scenario, the boundaries between parasites bearing different resistance allele lineages merely reflect meeting points between ripples, and these boundaries are expected to become blurred and disappear over time. Alternatively, the distinctive focal distribution could result from barriers to mixing and gene flow between parasite subpopulations.

How can the opposing “ripples on a pond” and “barriers to mixing” models be distinguished? One option might be to examine patterns of spread of resistance genes to other antimalarial drugs. If similar distribution patterns are observed at different resistance loci, then this may be indicative of common boundaries to gene flow. It will be extremely interesting to see if a pan-African analysis of dihydrofolate reductase (*dhfr*), which confers resistance to PYR, shows similar patterns. Furthermore, if boundaries between allelic lineages correspond to physical barriers such as mountain ranges or linguistic barriers between human populations, this further bolsters the “barriers to mixing” model.

Inferring the presence of barriers between populations using genes that are known to be under strong selection is fraught with problems [5]. Perhaps a more convincing approach might be to use the thousands of single nucleotide polymorphisms (SNPs) discovered by parasite re-sequencing projects [6] to infer parasite population structure. These can be scored using microarrays or short-read sequencing (Solexa), and the majority will not be influenced by selection. The power afforded by use of thousands of markers may reveal discontinuities in parasite populations that were not evident in smaller previous studies [7].

## Implications for Resistance Surveillance and Malaria Control

This new study provides a number of important lessons. First, it illustrates the practicality of routine measurement of drug resistance allele frequencies across malaria-endemic regions. Such measurement allows policy makers direct access to information required to make national drug policy decisions. This new study was done in a single laboratory, using samples from multiple countries, showing that centralization can provide a practical way to generate such databases. The alternative model—multiple regional laboratories generating such data—will be more expensive and less practical from a control quality standpoint. Notably, this work was done with quite simple molecular methods—hybridization on nylon membranes. Rapid SNP typing approaches now allow multiple resistance SNPs to be genotyped in a single assay at low cost [8], which can further streamline geographical mapping of multiple resistance determinants.

The distinctive differences observed between east and west Africa have clear clinical implications. The *dhps* alleles with two mutations (SGE) confer higher levels of resistance than those carrying single mutations (AGK and SGK) [9], suggesting that SDX-PYR treatment will be less effective in east than west Africa. Hence interventions that use SDX-PYR, such as intermittent preventative therapy for infants or pregnant women, might be expected to differ in efficacy depending on location.

Given that just one or two SNPs are required for resistance, and infected people may contain more than 10^11^ parasites [10], the finding that resistance alleles have only five origins is surprising, but consistent with limited numbers of origins observed at other drug resistance loci in malaria parasites [11] and some population genetic models [12]. This observation bodes well for current control efforts using artemisinin combination therapies. If resistance has relatively few independent origins, then simple strategies like combination therapy may be extremely effective in stalling resistance evolution [10], [13]. On the other hand, if resistance arises commonly, then such measures are more likely to be ineffective.

Finally, Roper and colleagues' study generates a testable hypothesis that malaria parasite populations in Africa are divided into regional subpopulations separated by barriers to gene flow. Further data are required to substantiate this hypothesis. If true, this hypothesis has important implications as it suggests that malaria control needs to be tailored to regional parasite subpopulations rather than to the continent as a whole.

## Abbreviations

*dhps*: dihydropteroate synthase
PYR: pyrimethamine
SDX: sulfadoxine
SNP: single nucleotide polymorphism
